# New Appliances & Things Medical

**Published:** 1911-08-05

**Authors:** 


### NEW APPLIANCES & THINGS MEDICAL

### SHELTERS AND CHALETS.

(Boulton and Paul, Ltd., Norwich.)

Messrs. Boulton and Paul send us an illustrated booklet describing their outdoor shelters, pavilions, bungalows, and chalets. We have been enabled, through the courtesy of professional friends who have erected this firm's shelters, to test the durability, comfort, and general excellence of the chalets, and we can testify to their uncommon merits from every point of view. Out-of-door existence is becoming more popular every year, and we know of no better way in which the average City man, who for a whole winter has been living in the vitiated $H_2SO_4$ impregnated atmosphere of a large town, can recuperate his health and enjoy the comforts of outdoor life than by buying one of these shelters, erecting it in his back garden or on any suitable site he can obtain, and sleeping in it. The prices quoted are reasonable considering the excellence of the materials used. For consumptive patients such shelters are almost indispensable, and probably do as much good as the more expensive sanatorium treatment. The glazed sun shelter described on p. 28 of the booklet is listed at £14—a ridiculously cheap price considering its attractiveness and usefulness. The price list is forwarded free on application.

